# Current practices in children with severe acute asthma across European PICUs: an ESPNIC survey

**DOI:** 10.1007/s00431-019-03502-9

**Published:** 2019-12-03

**Authors:** Shelley Boeschoten, Matthijs de Hoog, Martin Kneyber, Peter Merkus, Annemie Boehmer, Corinne Buysse

**Affiliations:** 1 Department of Pediatric Intensive Care Unit/Pediatric Surgery, Erasmus Medical Centre, Sophia’s Children Hospital, PO Box 2060, 3000CB Rotterdam, The Netherlands; 2 Department of Pediatrics, Division of Pediatric Intensive Care, University Medical Center Groningen, Groningen, The Netherlands; 3 Division of Respiratory Medicine, Department of Pediatrics, Radboud University Medical Centre Amalia Children’s Hospital, Nijmegen, The Netherlands; 4 Department of Pediatrics, Erasmus Medical Centre, Sophia’s Children Hospital and Maasstad Hospital, Rotterdam, The Netherlands

**Keywords:** Intensive care, Status asthmaticus, Severe acute asthma, Asthma guidelines, SCAMP

## Abstract

Most pediatric asthma guidelines offer evidence-based or best practice approaches to the management of asthma exacerbations but struggle with evidence-based approaches for severe acute asthma (SAA). We aimed to investigate current practices in children with SAA admitted to European pediatric intensive care units (PICUs), in particular, adjunct therapies, use of an asthma severity score, and availability of a SAA guideline. We designed a cross-sectional electronic survey across European PICUs. Thirty-seven PICUs from 11 European countries responded. In 8 PICUs (22%), a guideline for SAA management was unavailable. Inhaled beta-agonists and anticholinergics, combined with systemic steroids and IV MgSO_4_ was central in SAA treatment. Seven PICUs (30%) used a loading dose of a short-acting beta-agonist. Eighteen PICUs (49%) used an asthma severity score, with 8 different scores applied. Seventeen PICUs (46%) observed an increasing trend in SAA admissions.

*Conclusion*: Variations in the treatment of children with SAA mainly existed in the use of adjunct therapies and asthma severity scores. Importantly, in 22% of the PICUs, a SAA guideline was unavailable. Standardizing SAA guidelines across PICUs in Europe may improve quality of care. However, the limited number of PICUs represented and the data compilation method are constraining our findings.

**Table:** 

**What is Known:** • *Recent reports demonstrate increasing numbers of children with SAA requiring PICU admission in several countries across the world.* • *Most pediatric guidelines offer evidence-based approaches to the management of asthma exacerbations, but struggle with evidence-based approaches for SAA beyond these initial steps.* **What is New:** • *A large arsenal of adjunct therapies and 8 different asthma scores were used.* • *In a large number of PICUs, a written guideline for SAA management is lacking.*

**Electronic supplementary material:**

The online version of this article (10.1007/s00431-019-03502-9) contains supplementary material, which is available to authorized users.

## Introduction

Severe acute asthma (SAA), also known as status asthmaticus, is a severe or life-threatening asthma exacerbation, which does not respond to conventional treatment with inhaled short-acting beta-agonist (SABA) and systemic corticosteroids [1]. SAA has the potential to progress to respiratory failure and can be fatal [2]. SAA requiring admission to the pediatric intensive care unit (PICU) occurs in approximately 5–16% of all asthma hospitalizations [3–5]. Recent reports demonstrate increasing numbers of children requiring PICU admission in several countries across the continents [3, 5–7].

Most pediatric asthma guidelines offer evidence-based approaches to the management of asthma exacerbations (oxygen, inhaled beta-agonists, and systemic corticosteroids). However, they struggle with an evidence-based approach for SAA beyond these initial steps [8–10]. There is a broad arsenal of adjunct therapies in case the child is not responding to conventional treatment, but the evidence is often unclear, conflicting or absent in the context of PICU care in literature. Adjunct therapies that are often suggested in children with SAA are intravenous (IV) magnesium sulfate (MgSO_4_), IV theophylline, and IV salbutamol.

A recent meta-analysis suggested that IV MgSO_4_ is likely to be effective in avoiding hospitalization and improving airway patency in children with asthma exacerbation [11]. MgSO_4_ has the advantage of widespread availability, low cost, and minimal adverse effects. Another advantage of MgSO_4_ is the potential ability to block beta 1-adrenergic effects of salbutamol, which can prevent tachycardia due to IV salbutamol [12].

Methylxanthines (e.g., theophylline) are another option in the setting of SAA management, although not recommended in USA guidelines [13]. The British Thoracic Society (BTS) and the Scottish Intercollegiate Guidelines Network (SIGN) guideline state that aminophylline can be considered for children unresponsive to maximal doses of bronchodilators and steroids [14].

Severe bronchial constriction can prevent the delivery of inhaled beta-agonists to the distal airways in children with SAA; IV beta-agonists may cause more effective bronchodilation [9]. A single dose of IV salbutamol might be effective on time to discharge from hospital or PICU, duration of nebulization of salbutamol, and clinical asthma scores. But the evidence for a beneficial effect of a loading dose of IV salbutamol is lacking. There are several available pediatric asthma scores, but none of them has been sufficiently validated for children with SAA, especially in the PICU setting [15].

We aimed to characterize the current practices of children with SAA in European PICUs, in particular, adjunct therapies, use of an asthma severity score as a tool for evaluating the effectiveness of interventions, and use of a guideline for SAA management. We hypothesized that SAA management, especially beyond the initial treatment steps, varied widely between European PICUs. SAA exacerbations are associated with significant morbidity and can be fatal. In view of the life-threatening aspect of SAA and recent reports of increasing SAA PICUs admissions worldwide, this calls for a strict SAA management, to improve patient care and reduce PICU admissions. Furthermore, with this survey, we could identify future research questions and priorities across Europe, with the ultimate aim to optimize and standardize SAA management in European PICUs.

## Materials and methods

We conducted a cross-sectional electronic survey (using LimeSurvey, supplemental data 1) across all European Society of Paediatric and Neonatal Intensive Care (ESPNIC) members in 54 European countries (exact number of PICUs unknown). The survey was designed by the authors and instrumentalized in order to address all aspects of the research question and hypothesis. Questions were based on current published guidelines and themes emerging from recent literature on PICU treatment of life-threatening asthma in the PICU. The questions in our survey were clear and straightforward, with an appropriate length of the questionnaire and check questions. The survey was piloted on three PICU physicians for clarity and face validity. The questionnaire was written in English; it included 43 questions divided into 4 sections (general information, SAA in the PICU, medication, and follow-up) requiring 15 min on average to complete. Pediatric physicians working at a PICU in Europe were invited through the ESPNIC newsletter and by email to complete the questionnaire online [16]. We asked only one clinician to respond per PICU for feasibility reasons.

The survey was undertaken between November 2017 and April 2018. The database contains information regarding PICU characteristics, admission criteria, respiratory support, treatment, trend in SAA PICU admissions, and mortality. For feasibility reasons, we did not ask for the exact numbers of mortality and PICU admissions for children with SAA, but only a range in percentages. Each completed survey was entered into a password-protected database for analysis. Data were collected electronically in a case report form. Responses to the questionnaire were anonymous but site coded.

Data were descriptively analyzed. For regional differences, countries were divided into 3 geographical areas: Northern Europe (the Netherlands, Finland, and UK), Central Europe (France, Germany, and Belgium), and Southern Europe (Spain, Portugal, Turkey, Greece, and Italy), as described in previous studies [17]. Differences between regions and type of hospital were tested using the chi-square test. To assess differences in treatment by number of PICU beds available, the linear-by-linear chi-square association was used. All statistical analyses were carried out in SPSS version 21 (Chicago, IL, USA).

## Results

Thirty-eight physicians from 37 PICUs located in 11 European countries responded to the survey (Table 1). Of 1 PICU, 2 physicians responded to the survey, these responses were combined. The characteristics of the PICUs are described in appendix (Table 2). Severity of the SAA, the type of respiratory support, and the type of medication needed were the main primary indications for PICU admission (Table 3).

**Table 1:** Respondent characteristics

Respondents	Number of sites (%) ^a^
Country	
The Netherlands	7 (19)
France	6 (16)
Spain	6 (16)
Germany	5 (14)
UK	3 (8)
Portugal	3 (8)
Belgium	2 (5)
Turkey	2 (5)
Finland	1 (3)
Greece	1 (3)
Italy	1 (3)
Specialty	
Pediatric intensivist	35 (95)
Pediatric anesthesiologist	2 (5)
PICU experience (years)	
1–5	3 (8)
6–10	5 (14)
11–20	17 (46)
> 20	12 (32)

^a^38 respondents of 37 PICUs, 2 sites were combined

**Table 2:** PICU characteristics

PICU characteristics	Number (%, *N* = 37)
Hospital	
General hospital	1 (3)
University hospital	20 (54)
Children’s hospital	6 (16)
University children’s hospital	10 (27)
PICU beds	
1–10	14 (38)
11–20	19 (51)
21–30	3 (8)
> 30	1 (3)
PICU combined with ICU/NICU	10 (27)
Cardiac surgery	13 (35)
Overall PICU admissions per year	
< 250	1 (3)
251–500	17 (46)
501–750	8 (22)
751–1000	6 (16)
1001–1500	4 (11)
> 1500	1 (3)
Overall PICU mortality per year	
0–3%	16 (43)
4–5%	15 (41)
6–10%	6 (16)
Overall proportion IMV ^a^ per year	
< 25%	7 (19)
25–50%	16 (43)
50–75%	10 (27)
> 75%	4 (11)

^a^Invasive mechanical ventilation

**Table 3:** Primary indications for PICU admission in children with SAA

Admission criteria	*N* of PICU (*N* = 37)
Clinical signs ^a^	35
Persisting respiratory distress	28
Apparent fatigue	28
Altered level of consciousness	30
Respiratory support ^a^	37
NIV ^b^	34
HFNC ^c^	16
Invasive mechanical ventilation	33
Medication ^a^	25
Continuous nebulization	9
MgSO_4_ IV	1
SABA IV loading dose	6
SABA IV continuous infusion	23
* Independent of dose	22
* Dependent of dose	1

^a^At least one of these, multiple criteria were possible

^b^Non-invasive mechanical ventilation

^c^High-flow nasal cannula

**Fig. 1:**
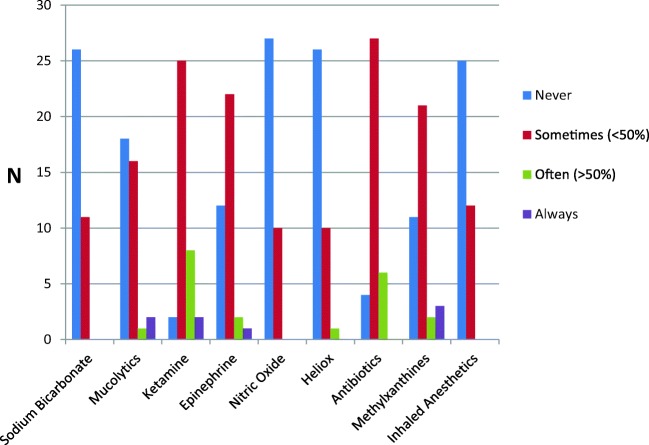
Adjunct therapies

The usual treatment for children with SAA was inhaled salbutamol combined with systemic steroids, an anticholinergic and IV MgSO_4_ (Table 4). Seven of 23 PICUs (30%) used a SABA IV loading dose. Different adjunct therapies were used in the PICUs (Fig. 1). The stepwise approach to the treatment of children with SAA differed significantly between all PICUs, with 25 different therapeutic options (supplemental Table 1). To assess the SAA severity, an asthma severity score was used in 18 PICUs (49%), with 8 different asthma scores used. The asthma score [18] was used most frequently (*n* = 7), followed by the respiratory rate, accessory muscle use, and decreased breath sounds score (RAD) (*n* = 4) [19]. Auscultation and accessory muscle use were represented in all asthma scores.

**Table 4:** Medication used in the treatment of SAA at the PICU

Medication	*N* of PICU (%, *N* = 37)
Inhalation with salbutamol	37 (100)
Systemic corticosteroid ^a^	37 (100)
IV	16 (43)
Oral or IV	21 (57)
Inhalation with an anticholinergic	35 (95)
IV MgSO_4_^b^	35 (95)
25 mg/kg	8 (23)
40 mg/kg	12 (34)
50 mg/kg	10 (29)
> 50 mg/kg	2 (6)
SABA IV loading dose ^c^	7 (19)
Salbutamol	5 (71)
Reproterol ^d^	2 (29)
< 10 mcg/kg	4 (57)
10–15 mcg/kg	3 (43)

^a^Route of administration of the systemic corticosteroids

^b^Dose of MgSO_4_

^c^Type of SABA

^d^Dose of reproterol

Twenty-nine PICUs (78%) used a written guideline to treat children with SAA in their hospital. Of these PICUs, 18 used a local guideline, 10 a national guideline (UK [14], the Netherlands [20], France (guideline not specified)), and 1 an international guideline (Greece [2]).

The use of invasive mechanical ventilation (IMV) differed widely between PICUs, from none up to 31–50% of all children with SAA being mechanically ventilated. Non-invasive mechanical ventilation (NIV) and high-flow nasal cannula (HFNC) was used > 50% of SAA cases in 10 and 12 PICUs, respectively (Table 5). Mortality due to SAA was 0–3% in 36 PICUs (4–5% in 1 PICU). The number of SAA PICU admissions per center ranged from 12 to 250 the last 5 years, with a median of 50 (IQR 20–100). Seventeen physicians (46%) reported an increasing trend in children with SAA admitted to the PICU (based on local database *n* = 9, personal impression *n* = 8). We did not ask for the exact numbers of PICU admissions for feasibility reasons. In 24 PICUs (65%), a follow-up visit was scheduled, mostly with a pediatric pulmonologist, within 6 weeks after PICU discharge.

**Table 5:** Respiratory support in children with SAA

Respiratory support	*N* of PICU (%, *N* = 37)
NIV ^a^ or CPAP ^b^	
< 25%	17 (46)
25–50%	10 (27)
51–75%	5 (14)
> 75%	5 (14)
HFNC ^c^	
< 25%	21 (57)
25–50%	4 (11)
51–75%	7 (19)
> 75%	5 (14)
Invasive mechanical ventilation	
Never	4 (11)
1–10%	24 (65)
11–30%	7 (19)
31–50%	2 (5)

^a^Non-invasive ventilation

^b^Continuous positive airway pressure

^c^High-flow nasal cannula

Asthma management by region, type of hospital, and number of PICU beds available is shown in Table 6. In the Southern European countries, an asthma severity score was used significantly more compared with Central and Northern European countries (*p* = 0.032).

**Table 6:** SAA management by region, number of PICU beds available, and type of hospital

	Use of a guideline	Use of an asthma score	Loading dose IV salbutamol	Increasing trend SAA PICU admissions
Region				
Northern (*n* = 11)	9 (82)	5 (46)*	1 (9)	6 (55)
Central (*n* = 13)	12 (92)	3 (25)	4 (31)	7 (54)
Southern (*n* = 13)	8 (62)	10 (77)	2 (15)	4 (36)
PICU beds				
1–10 (*n* = 14)	9 (65) *	10 (77)	2 (14)	5 (39)
11–20 (*n* = 19)	16 (84)	6 (32)	3 (16)	9 (50)
21–30 (*n* = 3)	3 (100)	2 (67)	1 (33)	3 (100)
> 30 (*n* = 1)	1 (100)	0 (-)	1 (100)	0 (-)
Type of hospital				
General (*n* = 1)	1 (100)	1 (100)	0 (-)	0 (-)
University (*n* = 20)	15 (75)	10 (50)	3 (15)	9 (47)
Children’s (*n* = 6)	5 (83)	2 (40)	2 (33)	3 (50)
University children’s (*n* = 10)	8 (80)	5 (50)	2 (20)	5 (56)
Total (*n* = 37)	29 (78)	18 (50)	7 (19)	17 (49)

Data presented as number (%)

*p < 0.05

## Discussion

This survey showed that most European PICUs were adherent to proven initial treatment for children with SAA. However, intensification with the use of adjunct therapies varied widely. A large arsenal of adjunct therapies was used despite even less evidence of efficacy. Gathering evidence for these strategies warrants further studies as a first step towards evidence-based clinical guidelines for SAA in children. Ketamine and antibiotics were frequently used in children with SAA across European PICUs. However, both ketamine and antibiotics have no effect on hospital admission rate, or need for mechanical ventilation [21, 22].

A loading dose of IV salbutamol can lead to relatively higher salbutamol levels immediately after infusion with a possible therapeutic advantage. Few small studies reported an effect on time to discharge from hospital or PICU, duration of nebulization of salbutamol, and clinical asthma scores after a bolus of IV salbutamol [23, 24] or terbutaline [25], [26, 27]. In some PICUs, methylxanthines were standard treatment for children with SAA, although again a Cochrane review in 2005 showed no reduction in symptoms, number of nebulized treatment, and length of hospital stay [28]. Evidence regarding mucolytic drugs and epinephrine for children with SAA is limited. Nitric oxide, heliox, sodium bicarbonate, and inhaled anesthetics are rarely used in the treatment of children with SAA.

There are several available pediatric asthma scores, but none of them has been sufficiently validated for children with SAA [15]. It is essential to adapt and validate an existing dyspnea score, specific for the intensive care setting. A validated asthma score is needed to study the efficacy of different interventions in the context of PICU care.

A striking observation is that 22% of the PICUs did not have written guidelines for the management of children with SAA. Asthma treatment protocols in the ED were effective in improving some areas of management, including drug therapy [29, 30]. However, the lack of evidence for many of the above-mentioned treatments hampers the development of an evidence-based guideline. In the vast majority of PICU practices, strong supportive evidence is lacking before a therapy is applied. However, proving the treatment’s effectiveness after implementing the therapy in practice is important. Overall, each PICU cares for a small number of heterogeneous patients with relatively rare diseases, which emphasizes the need for international collaboration. An integral approach to improve care could be realized through a standardized clinical assessment and management plan (SCAMP) and might provide evidence of the effectiveness of these therapies [8].

In most European PICUs clinical assessment, type of respiratory support and SABA IV continuous infusion were the main PICU admission criteria. HFNC as a PICU admission criterion is reported in half of the responding PICUs. HFNC is frequently used in children with SAA, which is in line with a previous study, where a significant increase in the use of HFNC since 2010 is reported [6]. Only 1 observational study evaluated the use of HFNC in children with SAA [31]. Mortality due to SAA was low, and the use of IMV was rare. Unfortunately, no association can be made between lack of written guidelines and mortality, or proportion of IMV, because these variables were reported in a range.

For one-third of the patients, no follow-up visit was scheduled for the child after PICU discharge. These children are at risk for more severe asthma exacerbations with PICU readmission [3] and undertreatment with inhaled corticosteroids. A long-term follow-up with targeted management seems a crucial preventive measure and warranted to prevent readmissions for SAA.

## Limitations

This survey was carefully designed and instrumentalized in order to address all aspects of the research question and hypothesis. However, it is possible that not all relevant themes have been addressed.

This survey might not be representative for all PICUs in Europe, since only 11 of the 54 ESPNIC countries participated and Eastern and Northern Europe were underrepresented. ESPNIC has more than 600 members from 54 countries, but the relation of members to the exact number of European PICUs who received the invitation for the survey cannot be determined. So, despite the existence of a European Society dedicated to pediatric intensive care and its professionals (ESPNIC), performing a survey among ESPNIC members turned out to be highly challenging. Although a survey among ESPNIC members is a practical approach, it might not capture the full range of European PICU practice. Finally, we must recognize although an ESPNIC SAA guideline is needed, adherence could also be challenging. Another limitation of this study, as with any survey, is a bias of self-reporting, which provides no validation of the accuracy of the data provided. We asked only one clinician to respond per PICU for feasibility reasons. This may have led to subjective information not representing local PICU policy.

## Conclusion

Inhaled beta-agonists and anticholinergics combined with systemic steroids and IV MgSO_4_ were central in the SAA treatment. Importantly, in 22% of the PICUs, written guidelines were not available. However, the limited number of PICUs represented and the data compilation method, with bias, are constraining our findings.

Future research questions should focus on the use of adjunct therapies in life-threatening SAA. In our opinion, the role of IV salbutamol including a loading dose should be determined. Also, the place of high-flow oxygen as well as NIV needs further study. Furthermore, validating an asthma severity score for use in a PICU setting as a tool for evaluating the effectiveness of interventions is essential. The ultimate aim is to establish an ESPNIC SAA guideline based on evidence and implementation of the guideline in European PICUs. Standard of care within European PICUs provides useful data which can lead to relevant research questions in future. Finally, priority should be to reduce the number of children with SAA requiring PICU admission by identifying risk factors for PICU admissions, better treatment at the ED and pediatric ward (e.g., loading dose IV salbutamol), and strict PICU admission criteria (e.g., continuous infusion of IV salbutamol > 1–2 mcg/kg/min, NIV).

## Electronic supplementary material

ESM 1 (DOCX 315 kb).

ESM 2 (DOCX 15 kb).

## Abbreviations

BTS: British Thoracic Society
ECMO: Extracorporeal membrane oxygenation
ED: Emergency department
ESPNIC: European Society of Paediatric and Neonatal Intensive Care
HFNC: High-flow nasal cannula
IV: Intravenous
MgSO_4_: Magnesium sulfate
PICU: Pediatric intensive care unit
PK: Pharmacokinetics
PTSD: Posttraumatic stress disorder
NIV: Non-invasive mechanical ventilation
RAD: Respiratory rate, accessory muscle use, decreased breath sounds score
RCT: Randomized controlled trial
SAA: Severe acute asthma
SABA: Short-acting beta-agonist
SCAMP: Standardized clinical assessment and management plan
SIGN: Scottish Intercollegiate Guidelines Network
