# Supplementary material for: Current practices in children with severe acute asthma across European PICUs: an ESPNIC survey
Source: Eur J Pediatr. 2019 Dec 3;179(3):455–61. doi: 10.1007/s00431-019-03502-9 (PMC7028840; doi:10.1007/s00431-019-03502-9)

**ESPNIC survey about children with severe acute asthma on a PICU in Europe**

Objective: to assess current practices in children admitted to a PICU in Europe with severe acute asthma

Methods: Members of the ESPNIC will be asked to fill in a questionnaire

**Introduction**

The current practices in children with severe acute asthma (SAA) who are admitted to a pediatric ICU vary greatly between PICUs. Through this online survey we would like to investigate the current practices concerning children with SAA admitted to a PICU in Europe.

The survey is coordinated by the Erasmus Medical Center, Rotterdam (the Netherlands). If you would like more information or may have problems when filling out the survey, please use our special emailadress: [static.studie@erasmusmc.nl](mailto:static.studie@erasmusmc.nl) or contact one of our researchers: Shelley Boeschoten, MD (Dept Intensive Care, Erasmus MC - Sophia Children's Hospital, Rotterdam, the Netherlands, [s.boeschoten@erasmusmc.nl](mailto:s.boeschoten@erasmusmc.nl).

The survey will take about 10 minutes to complete. The questions are only available in English.

**Part A: General information**

1. What is your country of work?

2. What is the name of your institution?

3. What type of hospital do you work in?

- General hospital
- University hospital
- Children’s hospital
- University children’s hospital

4. What is your profession?

- Pediatric intensivist
- Pediatric anesthesiologist
- Pediatrician
- Surgeon
- Nurse/nurse practitioner
- Other (specify____________________)

5. How many years of experience do you have working in a PICU (as a staff member and/or as a fellow)?

- 1-5 years
- 6-10 years
- 11-20 years
- >20 years

6. Is the PICU combined with an adult ICU or a neonatal ICU?

- Not combined
- Neonatal ICU
- Adult ICU
- Both adult and neonatal ICU

7. Does your PICU include cardiac surgery?

- No
- Yes

8. What is the number of pediatric ICU beds in your unit?

- 1-10
- 11-20
- 21-30
- >30

9. What is the average number of PICU admissions (until age 18 years) per year in your unit?

- <250
- 251-500
- 501-750
- 751-1000
- 1001-1500
- >1500
- Unknown

10. What is the average proportion of mechanically (invasive) ventilated pediatric patients (until age 18 years) per year in your unit?

- <25%
- 25-50%
- 50-75%
- >75%
- Unknown

11. What is the average mortality of all PICU patients (until age 18 years) per year in your unit?

- 0-3%
- 4-5%
- 6-10%
- >10%
- Unknown

**Part B: Severe acute asthma (SAA) in your PICU**

1. What are the PICU admission criteria for children with SAA in your PICU?

- Respiratory support (need for noninvasive ventilation, need for HFNC, need for invasive ventilation)
- Medication (SABA intravenously (if yes: independent or dependent of the dose (0-0.5 mcg/0.5-1.0/1.0-2.0/>2 mcg)), SABA loading dose, continuous/frequent nebulization with SABA, other)
- Other (specify__________________________)

2. What is the number of children with SAA aged 0-4 years admitted on your PICU per year?

- 0-5
- 6-10
- 11-20
- >20
- Unknown

3. What is the number of children with SAA aged 5-18 years admitted on your PICU per year?

- 0-5
- 6-10
- 11-20
- >20
- Unknown

4. Do you see an increasing trend in children with SAA admitted on your PICU?

- No
- Yes
- Unknown

*If Yes: can you say something about a possible cause for the increasing trend (e.g. due to change protocol, more asthma, lower threshold PICU admission)?

5. How many children with SAA (until age 18 years) are treated with noninvasive ventilation, high flow nasal cannula (HFNC) or continuous positive airway pressure (CPAP) on your PICU per year?

- <25%
- 25-50%
- 50-75%
- >75%
- Unknown

6. How many children with SAA (until age 18 years) are treated with invasive ventilation on your PICU per year?

- <25%
- 25-50%
- 50-75%
- >75%
- Unknown

7. Is there a guideline for children with SAA used in your PICU/hospital?

- No
- Yes

*If Yes: Is this a local or (inter) national guideline?

- Local guideline
- National guideline
- International guideline
- Unknown

** If National guideline; Which national guideline?

** If International guideline; Which international guideline?

8. Who is responsible for the treatment of the SAA patient in your PICU?

- Pediatric intensivist
- Pediatrician
- Pediatric pulmonologist
- Anesthesiologist
- Other (specify___________________________)

9. Do you use an asthma score to assess the severity of the asthma attack in your unit?

- No
- Yes

* If Yes: Which asthma score do you use?

- The Asthma Score (AS)
- Clinical Asthma Evaluation Score 2 (CAES-2)
- Asthma Severity Score (ASS)
- Pediatric Respiratory Assessment Measure (PRAM)
- Respiratory rate, accessory muscle use, decreased breath sounds (RAD)
- Other (Specify_____________________)
- Unknown

10. Based on which data/variable(s) do you decide to go to the next step in the treatment of SAA?

- Asthma score
- Blood gas
- Saturation/oxygen supply
- Clinical assessment
- Other (specify___________________________)

11. How many children with SAA (until age 18 years) are treated with extracorporeal membrane oxygenation (ECMO) on your PICU on average per year?

- 0-5%
- 6-10%
- 10-15%
- >15%
- Unknown

12. What is the average mortality of children with SAA (until age 18 years) per year in your unit?

- 0-3%
- 4-5%
- 6-10%
- >10%
- Unknown

**Part C: Medication in children with SAA**

1. What kind of nebulized short acting beta-2 agonist (SABA) do you use for children with SAA in your unit?

- Albuterol
- Levalbuterol
- Salbutamol
- Other (specify____________________________________________)
- We don’t use nebulized SABA in our PICU

2. What kind of systemic corticosteroids do you use for children with SAA in your unit?

- Prednisone
- Prednisolone
- Methylprednisolone
- Other (specify_____________________)

3. Do you give the systemic corticosteroids oral or IV?

- Oral
- IV
- Oral or intravenous, it differs

4. What is the usual dose of the systemic corticosteroids?

- <1 mg/kg/day
- 1 mg/kg/day
- 2 mg/kg/day
- >2 mg/kg/day

5. What is the maximum dose of the systemic corticosteroids?

- 40 mg/day
- 60 mg/day
- >60 mg/day

6. Are children with SAA nebulized with an anticholinergic (Ipratropiumbromide/Atrovent) in your hospital?

- No
- Yes

7. Are children with SAA treated with Magnesium Sulphate (MgSO_4_) in your hospital?

- No
- Yes

If Yes: How is MgSO4 administered?

- IV
- Inhaled

If Yes: What is the usual dose of MgSO4 administered in your hospital?

- 25 mg/kg
- 40 mg/kg
- 50 mg/kg
- >50 mg/kg
- Unknown

8. Is a loading dose of SABA intravenous part of the SAA treatment in children in your hospital?

- No
- Yes
- On indication
- Unknown

If Yes: What kind of SABA loading dose IV is used in your hospital?

- Salbutamol
- Terbutaline
- Other (Specify______________________)

If Yes: What is the usual dose for a loading dose of SABA IV in your hospital?

- <10 mcg/kg
- 10-15 mcg/kg
- >15 mcg/kg

If Yes: What is the maximum dose for a loading dose with SABA IV in your hospital?

- 250mcg
- 500mcg
- 750mcg
- 1000mcg
- No limit
- Unknown

9. What is the order of the medication for treating children with SAA in your hospital:

1 - Nebulized SABA

2 - Nebulized Anticholinergics

3 - Systemic corticosteroids

4 - MgSO4

5- Intravenous SABA

5- Methylxanthines

6 - Other (specify__________________________)

10. How often are adjunct therapies used in the treatment for SAA in children in your unit?;

Never (0)% Sometimes (<50%) Often (>50%) Always (100%)

Sodium Bicarbonate

Mucolytics (DNAse)

Methylxanthines (e.g. Theophylline, Aminophylline, Caffeine)

Inhaled anesthetics (e.g. Sevoflurane, Isofluorane, Halothane)

Ketamine

Epinephrine

Nitric Oxide

Heliox

Antibiotics

11. If you use other adjunct therapies, which one do you use in your unit?

**Part D: follow up**

1. Is every child (until age 18 years) admitted on your PICU with SAA scheduled for a follow-up visit?

- No
- Yes
- Unknown

*If Yes: After how many weeks is the follow-up visit scheduled?

- 0-6 weeks
- 6-12 weeks
- 3-6 months
- Unknown

** If Yes: In what kind of hospital will the visit take place (multiple options possible)?

- General hospital
- University hospital
- Children’s hospital
- University children’s hospital
- It differs in which hospital
- Other (specify_______________________)
- Unknown

** If Yes: who will see the child for follow-up (multiple options possible)?

- Pediatric intensivist
- Pediatric pulmonologist
- Pediatric anesthesiologist
- Pediatrician
- Surgeon
- Nurse/nurse practitioner
- Other (specify____________________)

2. Does your PICU have a specific database for all children with SAA admitted on your PICU?

- No
- Yes
- Unknown

Asthma scores (Eggink et al. Plosone):

1) Asthma score (AS)

2) Asthma Severity Score (ASS)

3) Clinical Asthma Evaluation Score 2 (CAES-2)

4) Pediatric Respiratory Assessment Measure (PRAM)

5) Respiratory rate, accessory muscle use, decreased breath sounds (RAD)

1, the AS (ref: Qureshi F, Pestian J, Davis P, Zaritsky A. Effect of nebulized ipratropium on the hospitalization rates of children with asthma. N Engl J Med 1998):


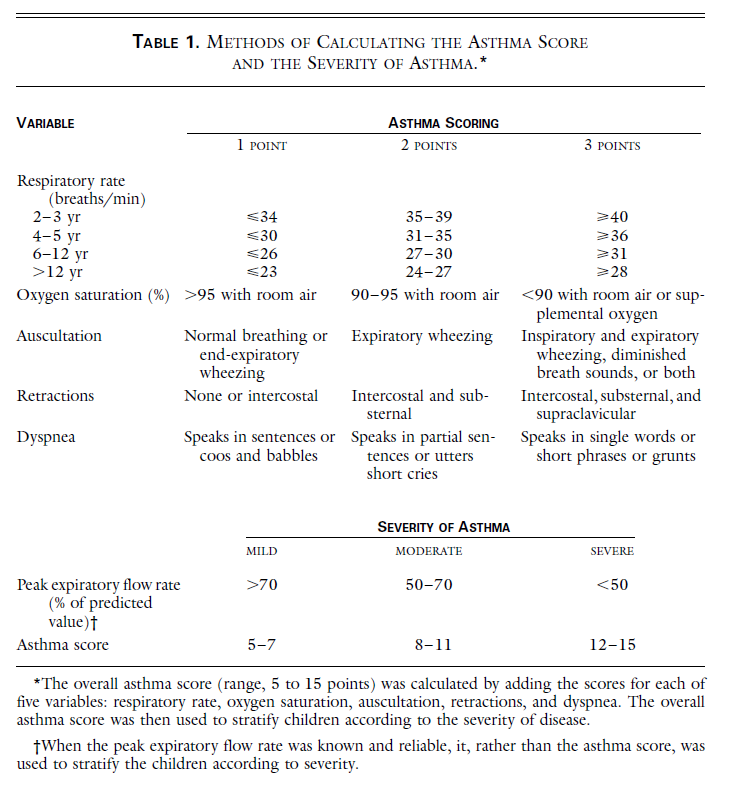


2, the ASS (ref : Janet Bishop et al. J Clin Epidemiol 1992):


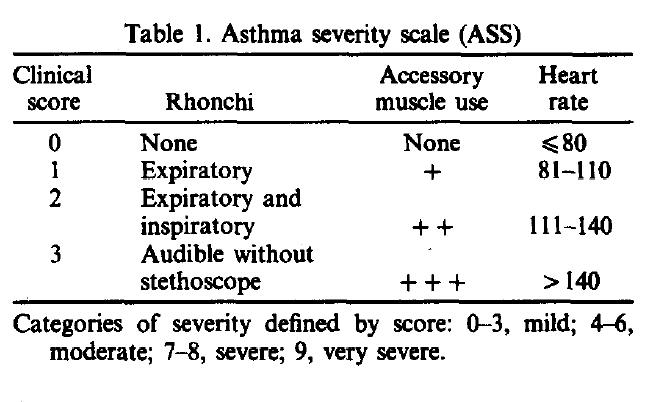


3, the CAES-2 (ref: Hurwitz et al. Clinical scoring, 1984, annals of emergency medicine):


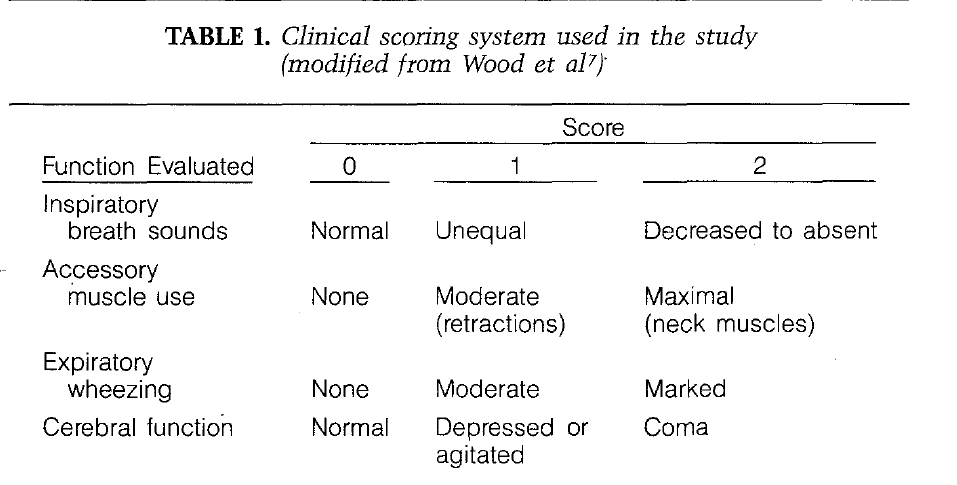


4, the PRAM (ref: Chalut, Ducharme Davis, The journal of pediatrics, 2000):


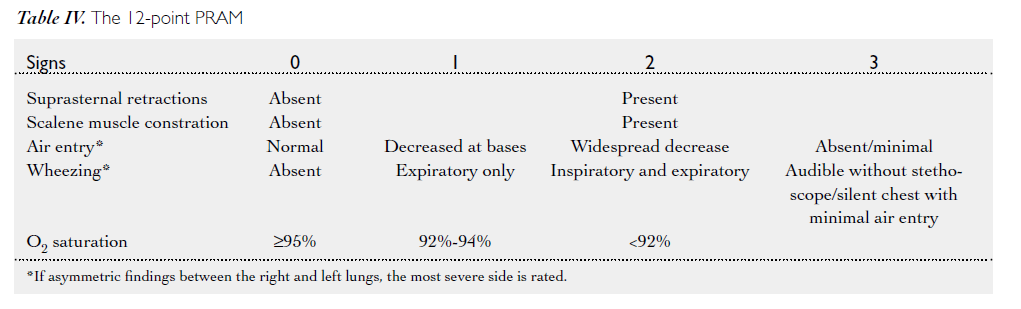


5, the RAD (ref: Arnold et al. 2011. Ann Allergy Asthma Immunol. RAD score):


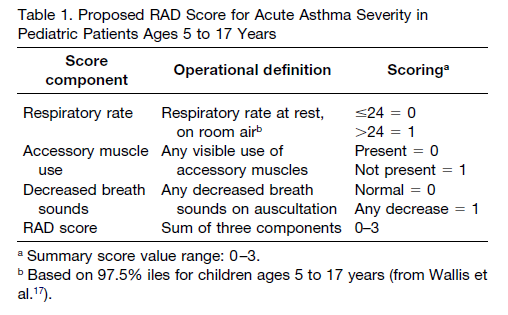

Supplement: Supplementary file 1 — (DOCX 315 kb). [file 431_2019_3502_MOESM1_ESM.docx]
