# Supplementary material for: Current practices in children with severe acute asthma across European PICUs: an ESPNIC survey
Source: Eur J Pediatr. 2019 Dec 3;179(3):455–61. doi: 10.1007/s00431-019-03502-9 (PMC7028840; doi:10.1007/s00431-019-03502-9)
Supplement: Supplementary file 2 — (DOCX 15 kb). [file 431_2019_3502_MOESM2_ESM.docx]

**Supplemental Table 1 - Order medication**

| Order medication | N | % |
| --- | --- | --- |
| Neb SABA - Neb Anticholinergic - Syst Cortico - MgSO_4_ - IV SABA - Methylxanthines - Other (Sodium Bicarbonate/Inh Anesthetics) | 8 | 21 |
| Neb SABA - Neb Anticholinergic - Syst Cortico - MgSO_4_ - IV SABA - Other (Sodium Bicarbonate/Inh Anesthetics) - Methylxanthines | 4 | 11 |
| Neb SABA - Syst Cortico - Neb Anticholinergic - MgSO_4_ - IV SABA - Other (Sodium Bicarbonate/Inh Anesthetics) - Methylxanthines | 2 | 5 |
| Neb SABA - Syst Cortico - Neb Anticholinergic - MgSO_4_ - IV SABA - Methylxanthines - Other (Sodium Bicarbonate/Inh Anesthetics) | 1 | 3 |
| Neb SABA - Neb Anticholinergic - Syst Cortico - IV SABA - MgSO_4_ - Methylxanthines - Other (Sodium Bicarbonate/Inh Anesthetics) | 2 | 5 |
| Neb SABA - Syst Cortico - Neb Anticholinergic - IV SABA - MgSO_4_ - Methylxanthines - Other (Sodium Bicarbonate/Inh Anesthetics) | 1 | 3 |
| Syst Cortico - Neb SABA - Neb Anticholinergic - IV SABA - MgSO_4_ - Methylxanthines - Other (Sodium Bicarbonate/Inh Anesthetics) | 1 | 3 |
| Neb SABA - Syst Cortico - IV SABA - Neb Anticholinergic - MgSO_4_ -Methylxanthines - Other (Sodium Bicarbonate/Inh Anesthetics) | 1 | 3 |
| Neb SABA - Syst Cortico - MgSO_4_ - IV SABA - Neb Anticholinergic - Methylxanthines - Other (Sodium Bicarbonate/Inh Anesthetics) | 1 | 3 |
| Neb SABA - Neb Anticholinergic - Syst Cortico - MgSO_4_ - Methylxanthines - IV SABA -Other (Sodium Bicarbonate/Inh Anesthetics) | 1 | 3 |
| Neb SABA - Syst Cortico - Neb Anticholinergic - MgSO_4_ - Methylxanthines - IV SABA - Other (Sodium Bicarbonate/Inh Anesthetics) | 2 | 5 |
| Neb SABA - Syst Cortico - MgSO_4_ - Neb Anticholinergic - Methylxanthines - IV SABA - Other (Sodium Bicarbonate/Inh Anesthetics) | 1 | 3 |
| Syst Cortico - Neb SABA - MgSO_4_ - Neb Anticholinergic - Methylxanthines - IV SABA - Other (Sodium Bicarbonate/Inh Anesthetics) | 1 | 3 |
| Neb SABA - Neb Anticholinergic - Syst Cortico - Methylxanthines - MgSO_4_ - IV SABA - Other (Sodium Bicarbonate/Inh Anesthetics) | 1 | 3 |
| Neb Anticholinergic - Syst Cortico - Neb SABA - MgSO_4_ - IV SABA - Other (Sodium Bicarbonate/Inh Anesthetics) - Methylxanthines | 1 | 3 |
| Neb SABA - Syst Cortico - MgSO_4_ - Neb Anticholinergic - IV SABA - Other (Sodium Bicarbonate/Inh Anesthetics) - Methylxanthines | 1 | 3 |
| Neb SABA - Syst Cortico - Neb Anticholinergic - IV SABA - MgSO_4_ - Other (Sodium Bicarbonate/Inh Anesthetics) - Methylxanthines | 1 | 3 |
| Neb SABA - Neb Anticholinergic - Syst Cortico - MgSO_4_ - Methylxanthines - Other (Sodium Bicarbonate/Inh Anesthetics) - IV SABA | 1 | 3 |
| Syst Cortico - Neb SABA - Neb Anticholinergic - MgSO_4_ - Methylxanthines - Other (Sodium Bicarbonate/Inh Anesthetics) - IV SABA | 1 | 3 |
| Neb SABA - Neb Anticholinergic - Syst Cortico - MgSO_4_ - Other (Sodium Bicarbonate/Inh Anesthetics) - Methylxanthines - IV SABA | 1 | 3 |
| Neb SABA - Syst Cortico - MgSO_4_ - IV SABA - Neb Anticholinergic -Other (Sodium Bicarbonate/Inh Anesthetics) - Methylxanthines | 1 | 3 |
| Neb SABA - Syst Cortico - Methylxanthines - MgSO_4_ - IV SABA - Other (Sodium Bicarbonate/Inh Anesthetics) - Neb Anticholinergic | 1 | 3 |
| Neb SABA - Syst Cortico - Methylxanthines - IV SABA - MgSO_4_ - Other (Sodium Bicarbonate/Inh Anesthetics) - Neb Anticholinergic | 1 | 3 |
| Neb SABA - Neb Anticholinergic - IV SABA - Syst Cortico - Methylxanthines - MgSO_4_ - Other (Sodium Bicarbonate/Inh Anesthetics) | 1 | 3 |
| Neb SABA - Syst Cortico - Neb Anticholinergic - IV SABA - Methylxanthines - MgSO_4_ - Other (Sodium Bicarbonate/Inh Anesthetics) | 1 | 3 |

Neb = nebulised, Syst Cortico = systemic corticosteroids
